# Prognostic impact of high levels of circulating plasmacytoid dendritic cells in breast cancer

**DOI:** 10.1186/s12967-016-0905-x

**Published:** 2016-05-28

**Authors:** Jithendra Kini Bailur, Brigitte Gueckel, Graham Pawelec

**Affiliations:** Department of Internal Medicine II, Centre for Medical Research, University Hospital Tübingen, Waldhoernlestr. 22, 72072 Tübingen, Germany; Radiology Clinic, Diagnostic and Interventional Radiology, University Hospital Tübingen, Tübingen, Germany; School of Science and Technology, College of Arts and Science, Nottingham Trent University, Nottingham, UK; Yale Cancer Center, Yale University School of Medicine, New Haven, CT USA; Division of Cancer Studies, Faculty of Life Sciences and Medicine, King’s College London, London, UK; Institute of Cancer Sciences, University of Manchester, Manchester, UK

**Keywords:** Breast cancer, T-cells, Plasmacytoid dendritic cells, Myeloid derived suppressor cells, Regulatory T-cells, Her-2

## Abstract

**Background:**

Identifying immune markers in blood that are informative for breast cancer patient survival would not only be useful for prognosis but might also provide mechanistic insights into processes facilitating survival.

**Methods:**

We phenotyped circulating plasmacytoid dendritic cells (pDCs), myeloid-derived suppressor cells (MDSCs) and regulatory T-cells in relation to T-cell responses to Her-2 in vitro in 75 untreated breast cancer patients 28–87 years of age at diagnosis.

**Results:**

Patients with later stage tumors had lower levels of circulating pDCs (p = 0.008). There was a positive association between 5-year survival and higher than median levels of circulating pDCs (p = 0.03). We confirmed that 5-year survival correlated with CD8+ but not CD4+ T-cell responsiveness to Her-2 peptides in this cohort of younger and older patients (p = 0.04). Including pDCs in the analysis of previously-established parameters revealed that patients who had a CD8+ T-cell response to Her-2 together with a low ratio of MDSCs:pDCs had 100 % 5-year survival. High levels of pDCs and the presence of a CD8+ T-cell response to Her-2 were independent positive survival indicators according to multivariate Cox analysis.

**Conclusions:**

Our new results suggest that circulating pDCs could be a positive prognostic indicator in breast cancer patients of all ages, together with the previously established CD8+ T-cell reactivity to Her-2 antigens in older patients only. These two prognostic indicators were independent and emphasize the important role of immunity in ensuring breast cancer patient survival, even in those not undergoing immunotherapy.

**Electronic supplementary material:**

The online version of this article (doi:10.1186/s12967-016-0905-x) contains supplementary material, which is available to authorized users.

## Background

Dendritic cells (DCs) play an important role in the presentation of antigens to T-cells, but also exert immunoregulatory activity [1]. There are two main subsets of DCs, monocytic DCs (mDCs) that are generally CD11c+, and plasmacytoid DCs (pDCs), also known as natural interferon-producing cells (IPCs), that are CD123+ (IL-α3R) [1, 2]. mDCs produce IL-12 and express Toll-like receptor (TLR)-1, -2, -3, -4, -5, -6, -7 and 8, whereas pDCs produce interferon-α and express TLR-7, -9 and 10 [3–6]. Many studies have used DCs to target cancer therapeutically [7, 8] but work on pDCs in the context of cancer immunity has focused more on their role in the tumor microenvironment than on whether their presence in the peripheral blood has any prognostic relevance. Increased levels of pDCs in breast cancer bone metastases and key roles in tumor growth have been reported in mice [9], and tumor-infiltrating pDCs have been negatively correlated with survival in some human cancers [10, 11] including breast cancer [12]. In melanoma, patients with smaller tumors have higher levels of blood pDCs [10] and numbers of circulating pDCs are reduced in cancer patients [13], suggesting that recruitment into the tumor may deplete these cells from peripheral blood. In melanoma, low levels of circulating pDCs have a negative correlation with survival [14]. On the other hand, high levels of circulating myeloid-derived suppressor cells (MDSCs), heterogeneous populations of immature dendritic cells, macrophages and granulocytes [15–17], have a negative impact on survival in different cancers [18, 19]. Together with regulatory T-cells (Tregs), these suppressive cells can form a formidable barrier preventing immune anti-tumor activity in cancer [20].

We have previously reported that peripheral T-cell reactivity to certain tumor-associated antigens (TAAs) in melanoma correlates with a survival benefit [21, 22]. Similarly, in breast cancer, the presence or absence of peripheral CD8+ T-cell responses to Her-2 peptides in vitro influences survival as shown in a cohort of elderly patients, whereas this was not the case for CD4+ T cell responses because these were present in almost all patients [23]. Compared to antibody therapy which is dependent on surface antigen expression, vaccination might induce better protection through the induction of T-cells recognizing cancer cells even with levels of surface Her-2 expression too low for antibody targeting and which are often designated “Her2-negative” in biopsy immunochemistry analyses [24]. An effective way to induce both TAA-reactive CD4+ and CD8+ T-cell responses is by using synthetic long peptides (SLPs) [25, 26]. Antigen presentation by pDCs could contribute to the induction of specific CD4+ and CD8+ T-cell responses [27, 28], but this would be contrary to the findings discussed above implying that high levels of pDCs in the tumor and low levels in the blood have a negative prognostic impact. Thus, the present study focuses on investigating the prognostic relevance of circulating antigen-presenting cells including total DCs, mDCs and pDCs separately, together with functional Her-2-reactive T-cells assayed in vitro, and an assessment of the impact of immunosuppressive cells on 5-year survival of breast cancer patients. This study goes beyond our previous work not only in examining pDCs but in extending the age range of the patients to include younger as well as elderly subjects.

## Methods

### Patients

Blood from 75 patients (28–87 years) from the University Hospital Tübingen was drawn between March and November 2009. Peripheral blood mononuclear cells (PBMCs) were isolated using standard Ficoll–Hypaque gradient centrifugation and cryopreserved because they were also intended to be used in multi-center studies requiring cell shipment. Patients were recruited at first diagnosis, prior to any treatment, and this was one of the main inclusion criteria. Also, in this study patients diagnosed with breast cancer from all age groups with any stage of the disease were included and no exclusion criteria were considered. Clinico-pathological data were available for almost all patients. Approval for the study was obtained from the Institutional Ethics Committee of University Clinic Tübingen (71/2009BO2) and a waiver of informed consent was granted.

### Phenotypic analysis of DCs, MDSCs and Tregs

PBMCs were thawed, washed and incubated with Gamunex and EMA, then stained with a cocktail of lineage (Lin) markers (CD3, CD19, CD56)-Brilliant Violet 605 (BioLegend, BD-Biosciences), CD14-Brilliant Violet711, CD11c-PE-Cy7 (BioLegend), CD45-V500, CD123-BV421, HLA-DR-PerCP-Cy5.5, CD15-FITC, CD11b-APC-Cy7, CD33-Alexa Fluor 700 (BD-Biosciences), and CD124-APC (R&D Systems) using EMA to identify dead cells.

To characterize Tregs, we used the same panel as before [23], staining PBMCs forCD3 (OKT3 supernatant) with Pacific Orange-conjugated secondary antibody (Invitrogen) followed by staining for CD4-Pacific Blue, CD45RA-Alexa Fluor-700, CD8-Peridinin-chlorophyll protein (PerCP), CD279-PerCP-Cy5.5, CD127-Alexa Fluor-647 (Bio legend), CD25-APC-Cy7 (BD Biosciences) and intracellular staining for FoxP3-PE (Bio legend). All samples were measured using a BD LSRII (BD Biosciences) immediately after staining.

### Detection of TAA-reactive T-Cells

PBMCs were thawed, washed, counted and re-suspended in X-Vivo 15 medium supplemented with IL-4 (5 ng/ml) and IL-7 (5 ng/ml) on day 0. On d1, pooled 15-mer Her-2 peptides (PepMix, JPT Technologies, Berlin) were added at 1 µg/ml to 1 × 10^6^ cells per culture. IL-2 (40U/ml) was added on d3. T-cells were harvested on d12 and re-stimulated (0.4–0.5 × 10^6^ cells/well) with 1 µg/ml Her2 peptides or left un-stimulated as a negative control for 12 h. Pepmixes of influenza nucleoprotein and matrix protein were used as positive controls. Golgi-plug (BD-Biosciences) was added at 1 µl/ml to prevent cytokine secretion. The cells were harvested, washed, incubated with Gamunex and EMA, fixed and permeabilized with Cytofix/Cytoperm (BD-Biosciences) before staining with CD3-Pacific Orange (Invitrogen), CD4-Pacific Blue, TNF-FITC, IL-2-Alexa-Fluor-700, IL-5-PE (BioLegend), CD8-APC-Cy7, IFN-γ-PE-Cy7 (BD-Biosciences), IL-10-APC (Miltenyi-Biotec) and IL-17-PerCP-Cy5.5 (eBioscience). After washing, the cells were immediately measured using a BD-LSR-II flow cytometer with FACS-Diva software (BD-Biosciences).

### Data analysis

Data were analyzed using FlowJo software (Tree-Star Inc.) after exclusion of duplicates using an FSC-area-versus-FSC-height/width plot for all flow cytometry datasets. To analyze DCs, CD45+ cells were gated on live cells, followed by gating the Lin(−) and CD14(−) cells. DCs were gated as HLA-DR(+) and then plasmacytoid [CD45(+)CD14(−)Lin(−)HLA-DR(+)CD123(+)] and myeloid [CD45(+)CD14(−)Lin(-)HLA-DR(+)CD11c (+)] DCs were gated from the HLA-DR(+) population. Further, MDSC populations were defined as Lin(−)CD14(+)HLA-DR(−) (MDSC-1) and Lin(−)CD14(+)CD124(+) (MDSC-2) as before [23]. CD45(+) cells were considered as the parental population for calculating the percentage of different subsets (see FACS plots and gating strategy in Additional file 1: Figure S1). Her-2-reactive T-cells producing cytokines in un-stimulated (negative control) samples compared to the stimulated samples were also gated as before using the same response criteria [23].

### Statistics

Chi square and Mann–Whitney U tests were performed to compare independent groups and Kaplan–Meier analysis (log-rank test) for survival, using GraphPad Prism 6. SPSS software was used to perform multivariate Cox analysis. A value of p < 0.05 was taken as statistically significant.

## Results

### Patients’ characteristics

The study comprised 75 (28–87 year-old) patients with a median age of 69 years at the time of blood draw. The clinico-pathological characteristics of the patients are summarized in Additional file 2: Table S1.

### DCs, MDSCs, Tregs and tumor characteristics

**Fig. 1:**
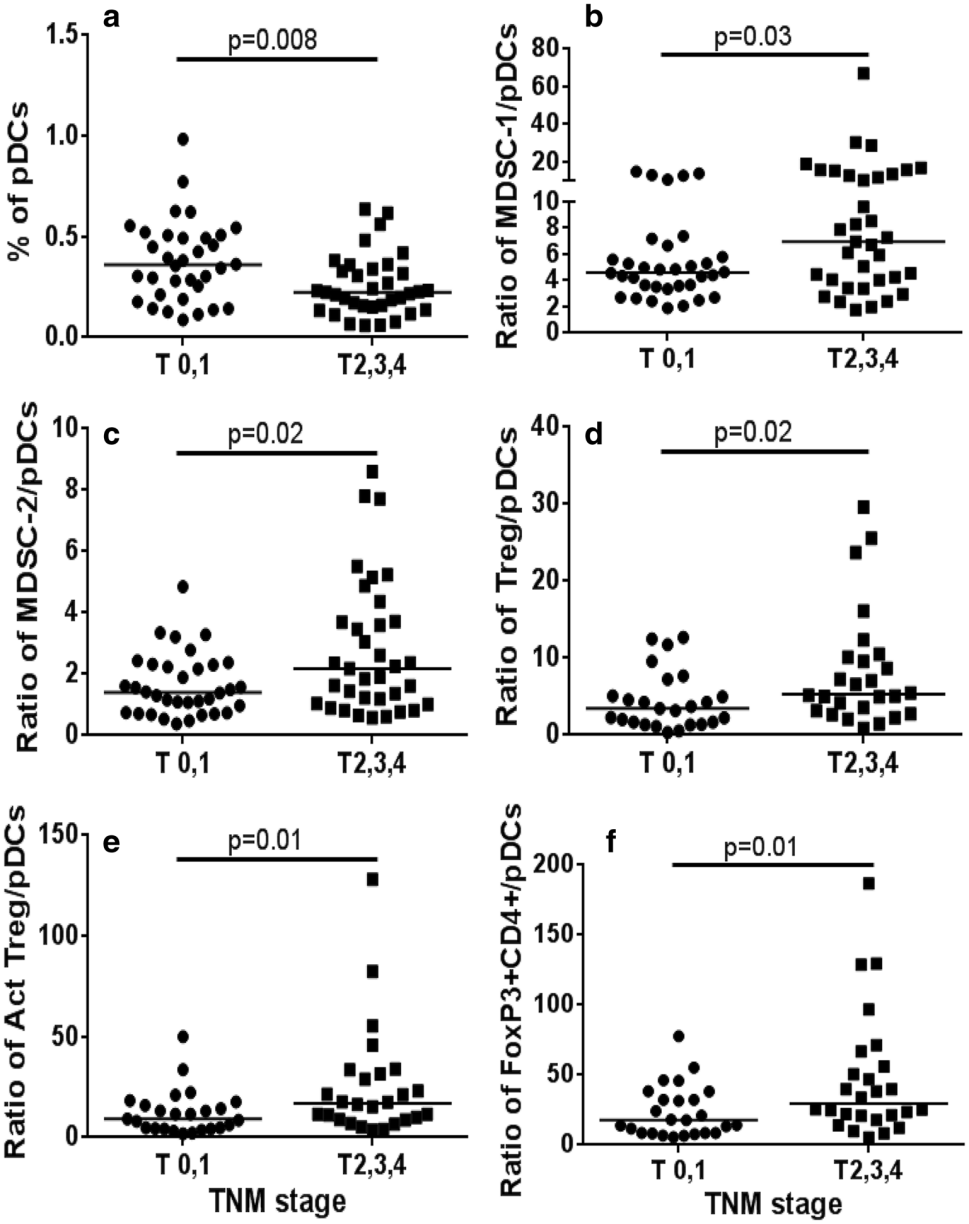
Distribution of DCs and ratio of DCs to immunosuppressive cells according to tumor stage. p values by Mann–Whitney U testfor (**a**) pDCs (**b**) MDSC/pDC (**c**) MDSC-2/pDCs (**d**) Treg/pDCs (**e**) aTreg/pDCs (**f**) FoxP3+ CD4+/pDCs

Neither the frequencies of total DCs nor mDCs in the peripheral blood were found to differ between patients at tumor stage 0, 1-versus-2, 3, 4 (data not shown). In contrast, patients at tumor stage 0 and 1 had significantly higher frequencies of pDCs than later stage patients (Fig. 1a, p = 0.008). Furthermore, the ratio of cells with the MDSC-1 phenotype to pDCs (Fig. 1b, p = 0.03), and the ratio of MDSC-2:pDCs (Fig. 1c, p = 0.02) were significantly higher in tumor stage 2, 3, 4. The other MDSC phenotypes were not informative (data not shown). The ratios of Tregs:pDCs (Fig. 1d, p = 0.02), activated Tregs:pDCs (Fig. 1e, p = 0.01) and FoxP3+ CD4+ T-cells:pDCs (Fig. 1f, p = 0.01) were also higher in tumor stage 2, 3, 4. Importantly, whether tumors were oestrogen receptor-positive or -negative, progesterone receptor-positive or -negative, or triple-negative did not influence the distribution of DCs or immunosuppressive cell types (data not shown).

### Level of DCs, immunosuppressive subsets and overall survival

**Fig. 2:**
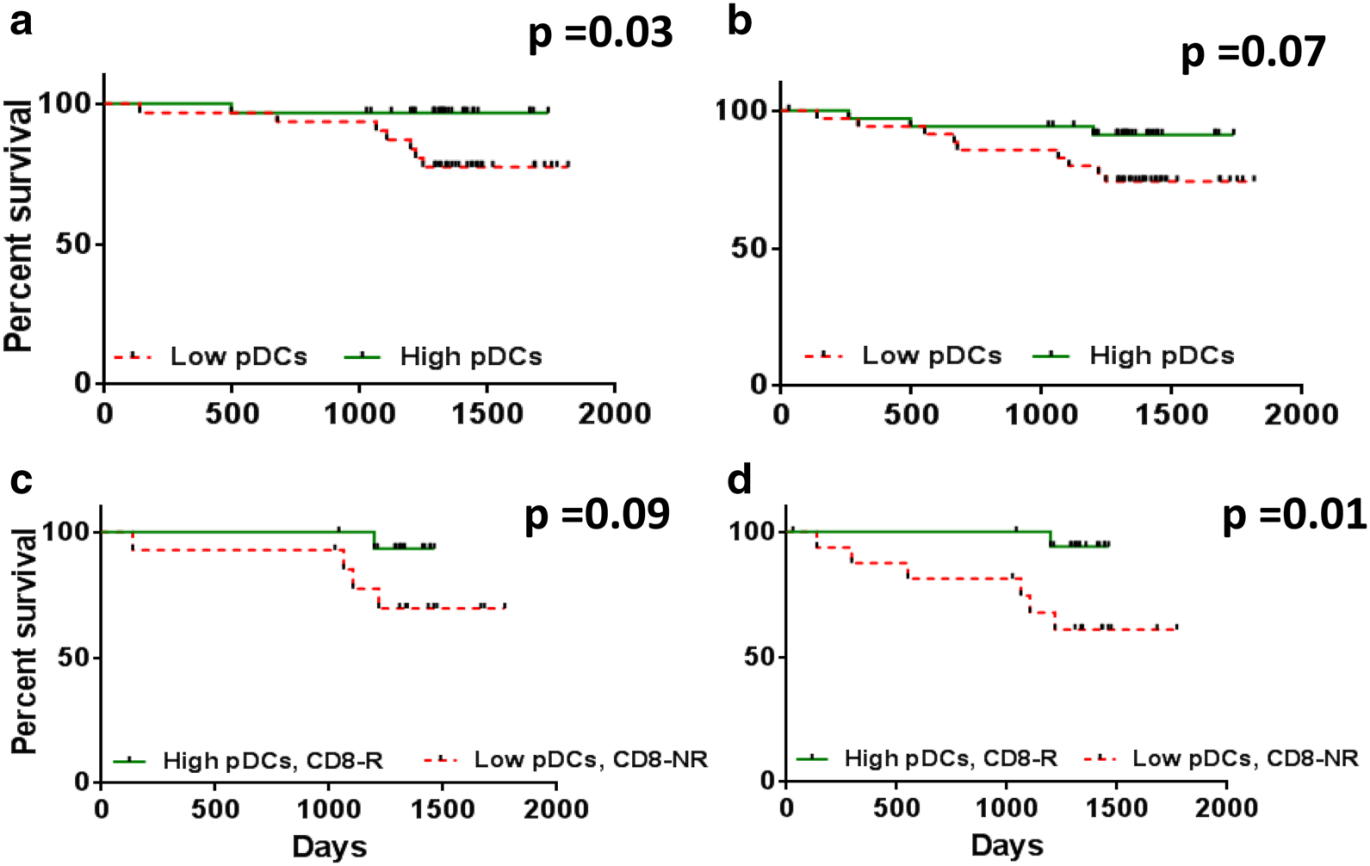
Kaplan–Meier survival analysis of non-metastatic (*left*) and all (*right*) patients according to the level of pDCs and for patients according to CD8+ T-cell response to Her-2 and level of DC subsets. Analysis of non-metastatic (**a**) and all patients (**b**) with low (≤median) versus high (>median) levels of DCs. Patients with CD8+ T-cell responses to Her-2 and high levels of pDCs compared to low levels of pDCs with no CD8+ T-cell response to Her-2, in **c** non-metastatic and **d** all patients

Kaplan–Meier analysis performed after stratifying patients according to their median frequencies of total DCs, mDCs or pDCs indicated significant differences in 5-year survival. For non-metastatic patients, there were no significant correlations between higher than median levels of total DCs or mDCs at baseline and survival (data not shown), butpatients with higher levels of pDCs did have significantly better survival (Fig. 2a, p = 0.03). Overall 5-year survival was as high as 97 % for patients with high levels of pDCs, whereas it was only 77 % for those with low levels. This survival advantage was also observed when all patients (metastatic and non-metastatic) were considered together, although no longer reaching significance (Fig. 2b, p = 0.07).

Although we did not observe any survival differences individually for the different immunosuppressive cells, there was some survival benefit when they were analysed together with the DCs. Thus, patients with a low ratio of MDSC-1:pDCs showed a trend towards better survival than those with a high ratio (p = 0.06) (Additional file 1: Figure S2A). This trend was also observed for the ratio of Tregs:DCs, where patients with low ratios of activated Tregs:total DCs (p = 0.07) (data not shown)and activated Tregs:mDCs (p = 0.07) (data not shown) tended to have better survival.

### Survival advantage of patients with a CD8+ T-cell response to Her-2

The great majority of patients possessed T-cells responding to Her-2 peptides in vitro (97 %). Memory CD4+ T-cell responses to Her-2 were detected in 89 % (65/73), whereas CD8+ T-cell responses were detected in only 39/73 (53 %) of patients. Patients were stratified according to whether they mounted a CD8+ T-cell response to Her-2 (irrespective of whether they had a CD4+ T-cell response) for survival analysis. Patients with a CD8+ T-cell response to Her-2had significantly better survival (p = 0.04) (data not shown) validating our earlier data in a smaller cohort [23].

### T-cell responses and tumor stage

We determined whether the proportion of patients possessing CD8+ T-cells responding to Her-2 differed depending on tumor stage. A significant decrease in the percentage of CD8+ T-cell responders to Her-2 was observed with increasing tumor stage, by Chi square testing (p = 0.02 for trend, Additional file 1: Figure S2B).

### Survival advantage of patients with a CD8+ T-cell response to Her-2, according to their frequency of DCs

Patients were grouped according to their T-cell responses to Her-2 and their level of DCs. Kaplan–Meier analysis showed that there was no longer a significant survival benefit for those with a CD8+ T-cell response to Her-2 if they also had high levels of total DCs, both in the case of metastatic and non-metastatic patients (data not shown). Taking mDCs separately, however, non-metastatic patients with a CD8+ T-cell response to Her-2 tended to have better survival even when they also had high levels of these cells (Additional file 1: Figure S2C, p = 0.06), whereas those with a high level of mDCs but no CD8+ T-cell response to Her-2 had the worst survival. This difference was significant when all patients were included in the survival analysis (p = 0.02). For pDCs, there was a tendency for non-metastatic patients with high levels of pDCs together with a CD8+ T-cell response to Her-2 to have better survival than those with no CD8+ T-cell response to Her-2 and low levels of pDCs (Fig. 2c, p = 0.09). The survival rate was 93 % in patients with a high level of pDCs together with a CD8+ T-cell response to Her-2 compared to 70 % in patients with no CD8+ T-cell response to Her-2 and low levels of pDCs. This survival advantage was also observed when all patients, both metastatic and non-metastatic, were considered together (Fig. 2d, p = 0.01), where patients with high pDCs and CD8+ T-cell responses to Her-2 had a 94 % 5-year survival compared to only 61 % for patients with no CD8+ T-cell response to Her-2 and low levels of pDCs. Further, similar advantages were observed for non-metastatic (Additional file 1: Figure S3A, p = 0.07) and all patients (Additional file 1: Figure S3B, p = 0.03) for the low ratio of mDC:pDC together with a CD8+ T-cell response to Her-2.

Kaplan–Meier analysis showed that metastatic patients had poorer survival, as did those not receiving radiotherapy and chemotherapy, as expected (Table 1). Multivariate Cox analysis showed that lack of CD8+ T-cell response to Her-2 had an independent impact on survival, in addition to no radiotherapy (Table 2, Model-1). As shown earlier, non-metastatic patients with high levels of pDCs had significantly better survival than those with low levels, in Model-2 (Table 2). When metastatic patients were not included in the multivariate Cox analysis and only four factors (T-cell response to Her-2, pDCs, chemotherapy and radiotherapy) were considered, there was an independent impact on survival for patients with high levels of pDCs in addition to a CD8+ T-cell response to Her-2.

**Table 1:** Kaplan–Meier analysis

Factor	N	% Dead (5 years)	p
Triple negative			0.57
Yes	13	23	
No	60	17	
Her-2 status			0.7
Neg	63	17	
Pos	9	22	
Metastasis			*<0.0001*
Yes	7	57	
No	67	15	
Radiotherapy			*<0.0001*
Yes	57	11	
No	17	47	
Hormonal therapy			0.08
Yes	60	17	
No	12	33	
Chemotherapy			*0.0006*
Yes	30	3	
No	42	31	
CD8 responseto Her-2			*0.04*
Yes	39	13	
No	34	26	
DCs			0.2
<Median	36	22	
≥Median	35	11	
mDCs			0.8
<Median	36	17	
≥Median	35	17	
pDCs			0.07
<Median	36	25	
≥Median	35	9	

Model 1 & 2 show the two different Multivariate analysis considering different significant factors, with and without metastasis

**Table 2:** Multivariate Cox analysis

Prognostic factor	N	Dead over 5 years (%)	Hazard ratio (95 % CI)	p
*Model-1*				
CD8 response to Her-2			0.198 (0.05–1.17)	*0.018*
Yes	37	11		
No	33	24		
Metastasis			3.647 (0.89–14.88)	0.071
No	32	15		
Yes	42	57		
pDCs			0.292 (0.07–1.17)	0.083
≤Median	36	25		
>Median	34	9		
Radiotherapy			0.107 (0.027–0.41)	*0.001*
Yes	57	11		
No	18	44		
Chemotherapy			<0.001 (<0.001–1.67e+130)	0.941
Yes	30	3		
No	42	31		
*Model-2*				
CD8 response to Her-2			0.145 (0.02–0.81)	*0.029*
Yes	34	12		
No	28	21		
pDCs			0.093 (0.011–0.809)	*0.031*
≤Median	31	23		
>Median	31	3		
Radiotherapy			0.091 (0.017–0.492)	*0.005*
Yes	53	9		
No	13	38		
Chemotherapy			<0.001 (<0.001–1.9e+157)	0.949
Yes	30	3		
No	34	26		

Model 1 & 2 show the two different Multivariate analysis considering different significant factors, with and without metastasis

### DCs, immunosuppressive subsets and cellular responses to Her-2

**Fig. 3:**
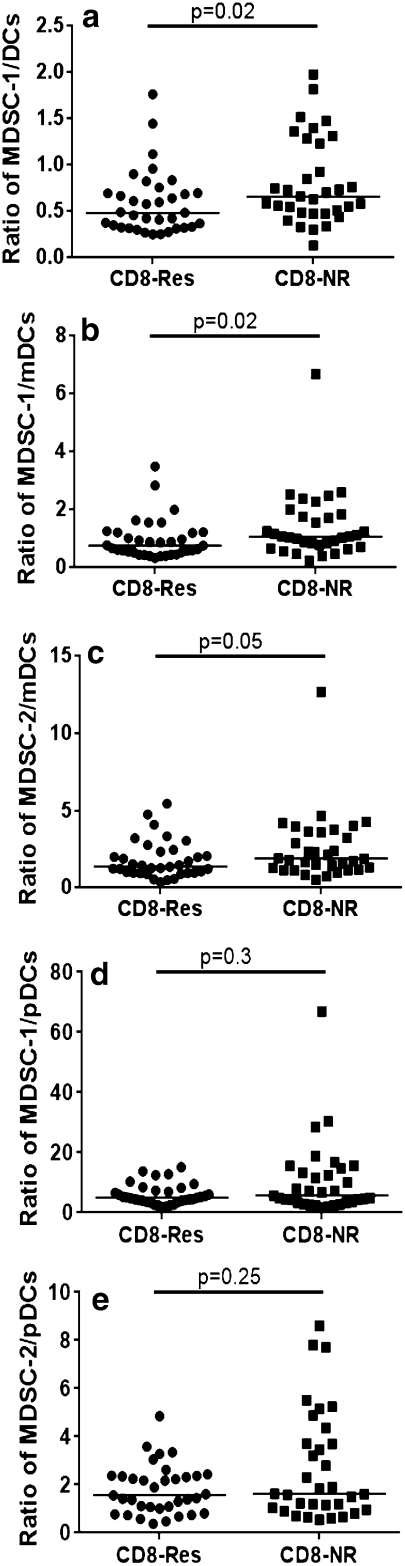
Percentage of immune subtypes in CD8-Res and CD8-NR. Patients with CD8+ T-cell responses to Her-2 (CD8-Res) compared to patients with no CD8+ T-cell responses (CD8-NR) with the ratio of **a** MDSC-1 to DCs (p = 0.02), **b** MDSC-1 to mDCs (p = 0.02), **c** MDSC-2 to mDCs, **d** MDSC-1 to pDCs and **e** MDSC-2 to pDCs

To investigate the influence of the presence of cells with a suppressive phenotype on the ability of patients’ PBMCs to mount a CD8+ T-cell response to Her-2, the frequencies of DCs, MDSCs and Tregs were grouped according to the presence or absence of the T-cell response. Patients without CD8+ T-cell responses to Her-2 had significantly higher levels of MDSC-1 (p = 0.01), activated Tregs (p = 0.03) and FoxP3+/CD4+ T-cells (p = 0.03) as well as a trend for MDSC-2 (p = 0.08) (data not shown). Patients with no CD8+ T-cell response to Her-2 had significantly higher ratios of MDSC-1:DCs (Fig. 3a, p = 0.02) and MDSC-1:mDCs (Fig. 3b, p = 0.02) again with a trend for MDSC-2:mDCs (Fig. 3c, p = 0.05). No differences were observed for ratios of MDSC-1:pDCs and MDSC-2:pDCs (Fig. 3d, e). Similar trends were observed for the ratio of Tregs:DCs (data not shown).

### Prognostic relevance of DCs together with MDSCs, regulatory T-cells and CD8+ T-cell responses to Her-2

**Fig. 4:**
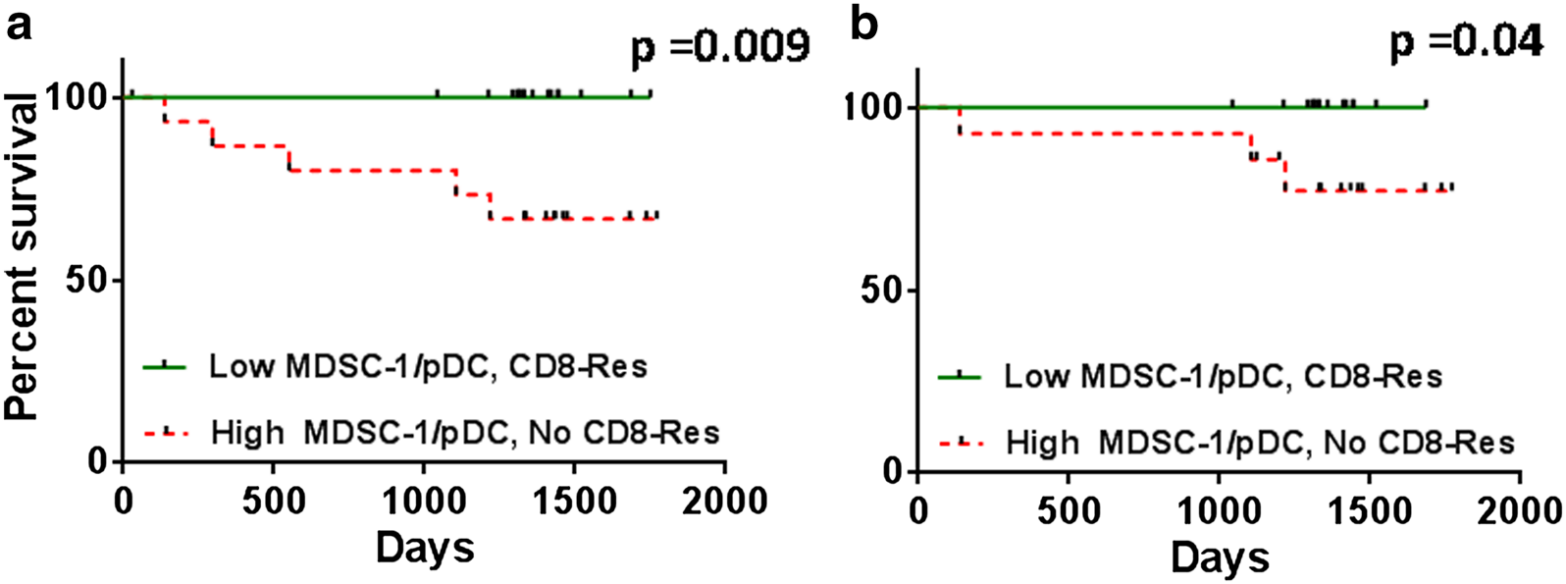
Kaplan–Meier survival analysis of all (*left*) and non-metastatic (*right*) patients according to CD8+ T-cell responses to Her-2 and ratios of MDSC-1 to pDCs. Kaplan–Meier analysis for patients with CD8+ T-cell responses to Her-2 and low ratios of MDSCs to pDCs compared to high ratios of MDSCs to pDCs with no CD8+ T-cell response to Her-2, in **a** all and **b** non-metastatic patients

Because pDCs emerged in this analysis as having a significant impact on survival, we next analyzed associations between DCs and different immunosuppressive cells, in relation to T-cell responses to Her-2 peptides. After calculating the ratio of MDSCs:pDCs, we grouped the patients according to whether or not they mounted a CD8+ T-cell response to Her-2. Kaplan–Meier analysis showed that patients with a CD8+ T-cell response to Her-2 together with a low ratio of MDSC-1:pDCs had a very significantly better survival (Fig. 4a, log rank test: p = 0.009) and that there was an early impact on survival (Breslow test: p = 0.009) with 100 % survival compared to those with a high ratio of MDSC-1:pDCs and no CD8+ T-cell responses to Her-2. This strong survival advantage was still present when only non-metastatic patients were considered (Fig. 4b, p = 0.04). In contrast, no survival association was observed for levels of MDSC-2 and total DCs. Finally, all patients with a CD8+ T-cell response to Her-2 and with a low ratio of Tregs:pDCs (Additional file 1: Figure S4A, p = 0.03), activated Tregs:pDCs (Additional file 1: Figure S4B, p = 0.03) or FoxP3+ CD4+ T-cells:pDCs (Additional file 1: Figure S4C, p = 0.03) survived for the 5 year follow-up. Similar survival benefits were observed for total DCs and mDCs as well.

## Discussion

DCs are recognized as important players in cancer immunotherapy and pDCs have emerged as potential vectors for immunotherapy [27, 29]. So far, to the best of our knowledge, there have been no studies correlating the levels of circulating DCs with clinical outcome in breast cancer. In addition, no study has investigated the relationships between DCs and immunosuppressive cell subsets and their influence on T-cell responses to TAA in breast cancer. We previously showed that older patients having low levels of MDSCs and CD8+ T-cell responses to Her-2 survived longer than those with high levels of MDSCs and no CD8+ T-cell responses to Her-2. Here, we extend and validate these results in a larger cohort also including younger patients and for the first time report a leading role of circulating pDCs in survival correlates.

Recent studies in mice have shown that pDCs can effectively induce anti-tumor CD8+ T-cell responses [30], and that pDCs efficiently cross-present TAA to trigger T-cell responses [28]. In the present study, we asked if there was any correlation between survival, patients’ in vitro T-cell responses to Her-2 peptides, and frequencies of circulating DCs. We found that 97 % of patients mounted CD4+ T-cell responses to Her-2, but only half had CD8+ responses, which associated positively with 5-year overall survival. Here, we sought clinicopathological parameters distinguishing those patients with CD8+ T-cell responses from those without, as well as correlates with the impact of DCs. Increasing tumor stage was identified as negatively associating with the frequency of anti-Her-2 CD8+ T-cell responses. Although no differences were observed in the levels of total DCs and mDCs between tumor stage 0, 1 and 2, 3, 4, the frequencies of peripheral pDCs were lower in patients with larger tumors. This may suggest sequestration of pDCs at the tumor site. Similar findings have been reported by others in an earlier study on melanoma, where the migratory profile of pDCs together with their frequency suggested their capture at the tumor site and draining lymph node, resulting in depletion of circulating pDCs [10]. We found that the ratios of different immunosuppressive subsets to pDCs were higher in tumor stage 2, 3, 4 patients, reflecting low pDC frequencies at larger tumor stage and indicating a possible indirect role played by the immunosuppressive cell subsets on DCs. Kaplan–Meir analysis showed that there was a survival advantage for patients with high levels of pDCs and CD8+ T-cell responses to Her-2 compared to patients with low levels of pDCs and no CD8+ T-cell response to Her-2 peptides. Similar results were observed for patients with a low ratio of mDCs:pDCs and a CD8+ T-cell response to Her-2. From the multivariate Cox analysis, it was concluded that high levels of pDCs and the presence of CD8+ T-cell responses to Her-2 had an independent effect on survival.

Although there are many studies showing the impact of Tregs and MDSCs in cancer, their relationship with total DCs, pDCs and mDCs, TAA-specific T-cell responses, and clinical outcome has been largely unexplored. In our study, a trend towards better survival was observed in patients with a low ratio of immunosuppressive cells to DC. Previous studies have shown that Tregs can suppress pDCs by forming aggregates [31] and that mDCs are also sensitive to Tregs [32]. In the present study, consistent with this notion, patients with CD8+ T-cell responses to Her-2 and low Tregs:pDCs ratios had better survival compared to those with no CD8+ T-cell response to Her-2 and a higher ratio of Tregs:pDCs, suggesting a clinically relevant suppressive role of Tregs. Also, we observed that patients with no CD8+ T-cell response to Her-2 had significantly higher ratios of MDSC-1:total DCs, MDSC-1:mDCs and MDSC-2:mDCs, indicating that those with no in vitro CD8+ T-cell response to Her-2 probably had low levels of APCs. Importantly, every patient exhibiting a CD8+ T-cell response to Her-2 together with low ratios of MDSC-1:pDCs was still alive at 5 years. Furthermore, we observed a high ratio of MDSCs:pDCs in patients with larger tumor burden, also indicating a possible negative impact on the maturation of myeloid cells with increasing disease stage. MDSCs not only impair T-cell and NK cell function, but also DC-vaccine quality as reported in one study, where it was shown that high levels of MDSCs in DC cultures could affect the co-stimulatory molecules CD80 and CD86, and important molecules like CD1a and DC-sign [33]. In another study by other investigators, patients with a high ratio of DCs:MDSCs responded more favorably to high-dose IL-2 [34], indicating the potential general importance of high levels of DCs and low levels of MDSCs.

Few studies have focused on the importance of pDCs and their influence on mDCs and vice versa [35–37]. We observed that patients with low ratios of mDCs:pDCs, who also had a CD8+ T-cell response to Her-2, had better survival compared to those with high ratios of mDCs:pDCs and no CD8+ T-cell response to Her-2, again indicating the importance of pDCs. In our study, patients with high levels of mDCs and a CD8+ T-cell response to Her-2 had better survival compared to those with high levels of mDCs and no CD8+ T-cell response to Her-2. This might be due to defective mDCs lacking efficient antigen presentation capacity, which might in turn be due to the lack of sufficient pDCs in these patients that produce type I IFNs indirectly affecting the activation of mDCs. It is known that activated pDCs can stimulate adjacent mDCs by the production of type I IFNs that could enhance their ability to cross-prime CD8+ T-cells. This notion is supported by mouse studies showing that type I IFNs play an important role in the induction of anti-tumor responses [4, 38].

A previous clinical study on metastatic melanoma showed that activated pDCs injected into lymph nodes resulted in the induction of TAA-specific CD8+ T-cells, some of which had high functional activity. From the retrospective analysis in that study, it was observed that patients treated with autologous TLR-activated and tumor antigen-loaded pDCs had increased survival compared to patients treated with chemotherapy [27]. Also, I-IFNs derived from pDCs are known to regulate T cell function, which includes long term T-cell survival and memory Th1 polarization, CD8+ T-cell cytotoxicity and IFN-γ production [39]. pDCs exert their effect snot only on T-cells but on NK cells as well, and they are known to increase NK cell-mediated cytotoxicity and IFN-γ production [40]. Our findings in the current study indicate an important role of Her-2-specific T-cells in patients having higher levels of circulating pDCs. We observed either CD4+ T-cell responses to Her-2, or both CD4+ and CD8+ T-cell responses, in almost all patients, although the majority of tumors had low expression of this molecule according to routine pathology (Her-2-0, 1, 2). This is consistent with the benefit of Her-2 vaccination that patients may experience even when their resected tumors are classified as having low or no expression of Her-2, as reported in some studies [41, 42].

## Conclusions

Our results from the present study emphasize the importance of the presence of high levels of circulating pDCs, low levels of immunosuppressive cells and the presence of CD8+ T-cell responses to Her-2 in predicting a favorable outcome in breast cancer patients both for all patients, and when only non-metastatic patients were considered. High levels of pDCs in blood, particularly in non-metastatic patients might reflect that they have not been sequestered by the tumor, hence there might be more pDCs available in the lymph node for TAA presentation, which in turn could enhance the induction of specific T-cells. On the other hand, the finding that high levels of pDCs and the presence of a CD8+ T-cell response to Her-2 were independent positive survival indicators according to multivariate Cox analysis suggests that mechanisms other than antigen presentation are responsible for the positive association of higher levels of circulating pDCs with survival in breast cancer. Regardless of the reason for these findings, the major observation from this study remains that circulating pDCs are positive prognostic indicators. Defining blood immune biomarkers informative for survival could not only enable prediction of an individual patient’s disease course without the need for biopsies, with all their limitations (limited access, unpleasant, potential triggering metastasis), but also provide information on the mechanisms mediating more successful cancer control by the immune system. Thus, measuring blood pDCs could represent part of a relatively simple blood test facilitating personalized interventions specifically tailored to the immune capacity of each individual patient.

## Additional files

10.1186/s12967-016-0905-x Representative FACS plots and additional results.
10.1186/s12967-016-0905-x Patients clinico-pathological parameters.

## Abbreviations

Her-2: human epidermal growth factor receptor-2
FoxP3: forkhead box P3
IL: interleukin
DCs: dendritic cells
MDSC: myeloid derived suppressor cell
TAAs: tumor associated antigens
aTreg: activated regulatory T-cells
MP: matrix protein
NP: nucleoprotein
