# Supplementary material for: Prognostic impact of high levels of circulating plasmacytoid dendritic cells in breast cancer
Source: J Transl Med. 2016 May 28;14:151. doi: 10.1186/s12967-016-0905-x (PMC4884426; doi:10.1186/s12967-016-0905-x)
Supplement: Supplementary file 1 — 10.1186/s12967-016-0905-x Representative FACS plots and additional results. [file 12967_2016_905_MOESM1_ESM.pptx]

## Slide 1
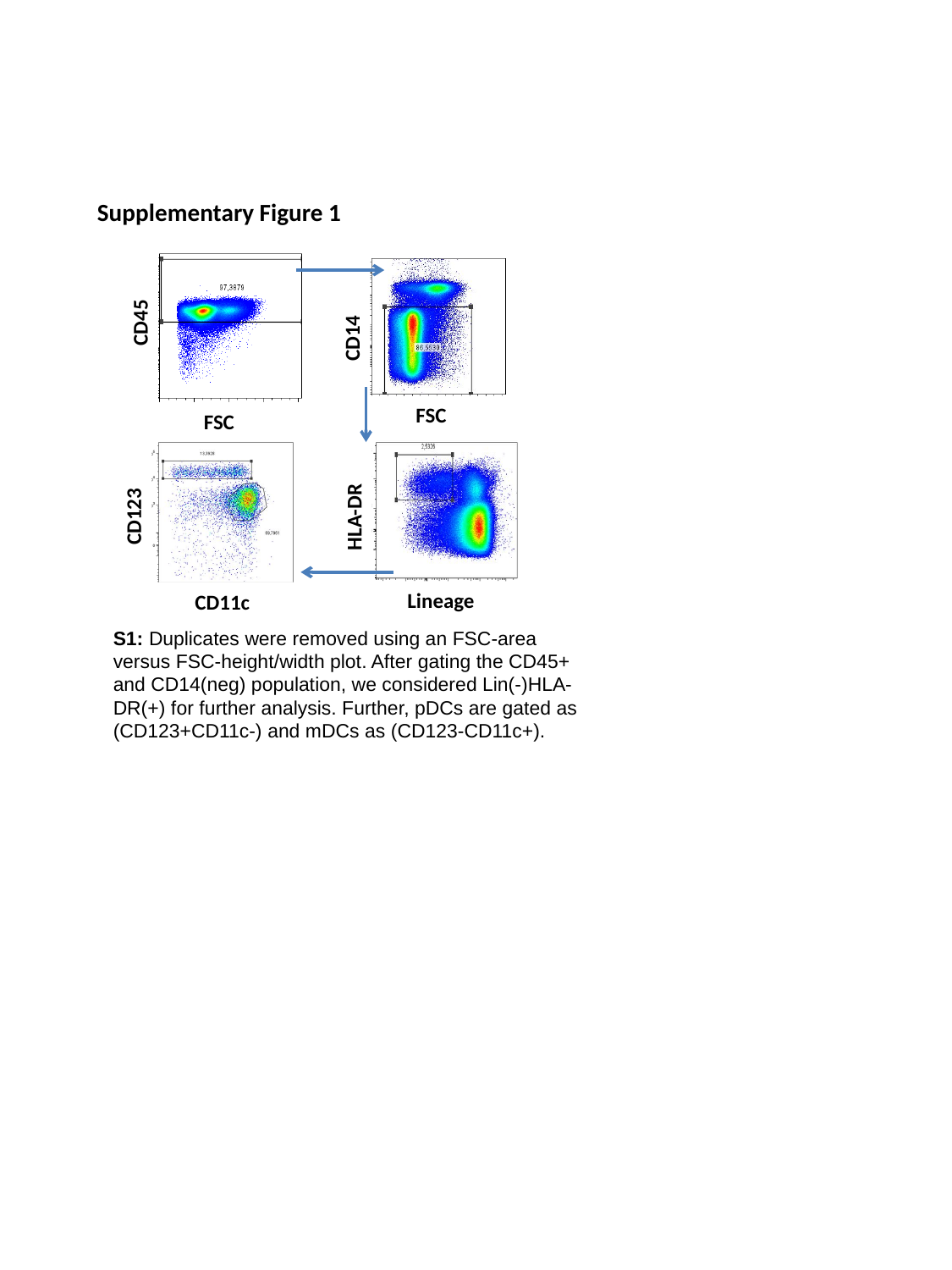

Supplementary Figure 1
CD45
CD14
 FSC
 FSC
CD123
HLA-DR
 Lineage
 CD11c
S1: Duplicates were removed using an FSC-area versus FSC-height/width plot. After gating the CD45+ and CD14(neg) population, we considered Lin(-)HLA-DR(+) for further analysis. Further, pDCs are gated as (CD123+CD11c-) and mDCs as (CD123-CD11c+).

## Slide 2
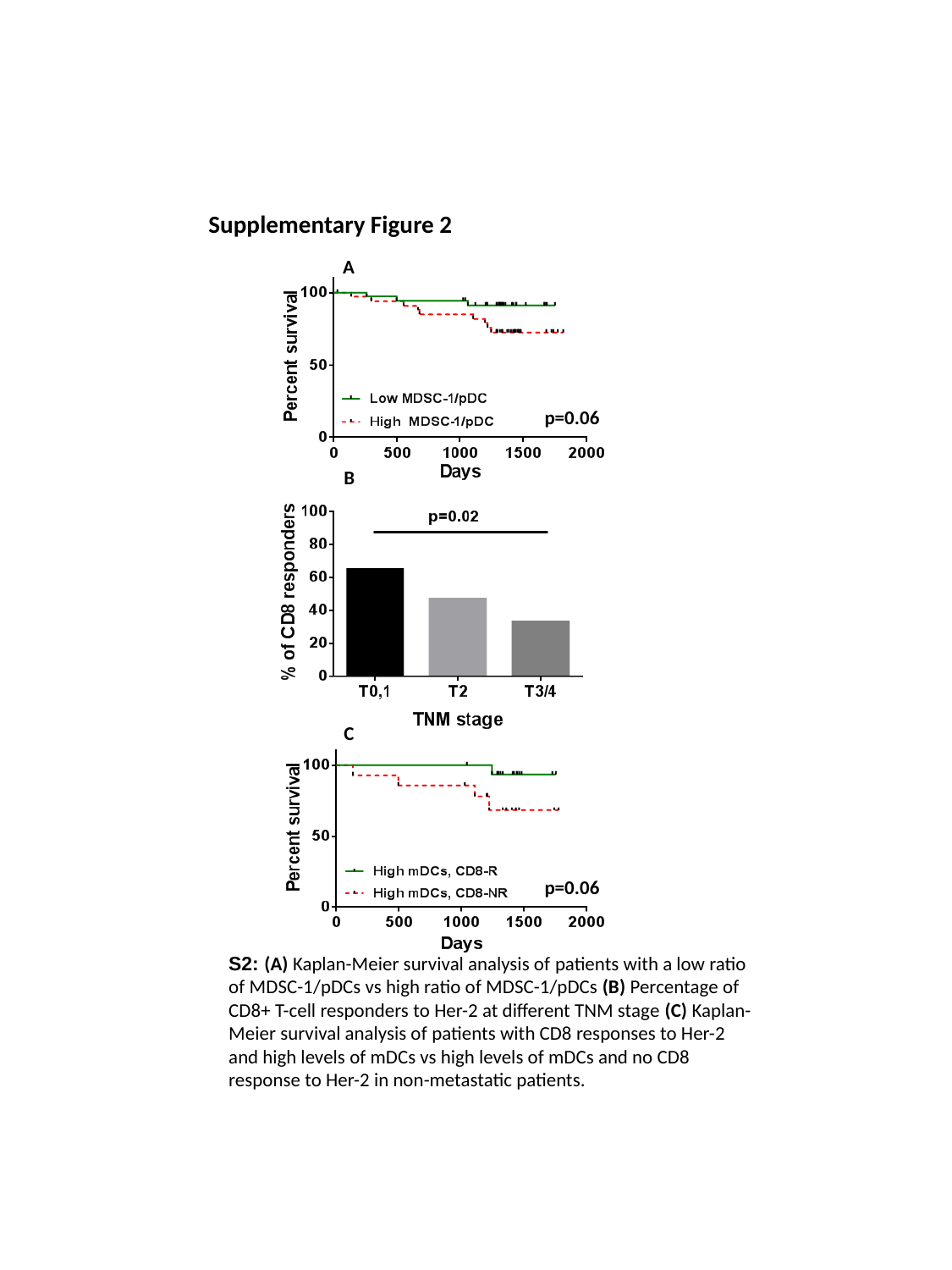

Supplementary Figure 2
A
p=0.06
B
C
p=0.06
S2: (A) Kaplan-Meier survival analysis of patients with a low ratio of MDSC-1/pDCs vs high ratio of MDSC-1/pDCs (B) Percentage of CD8+ T-cell responders to Her-2 at different TNM stage (C) Kaplan- Meier survival analysis of patients with CD8 responses to Her-2 and high levels of mDCs vs high levels of mDCs and no CD8 response to Her-2 in non-metastatic patients.

## Slide 3
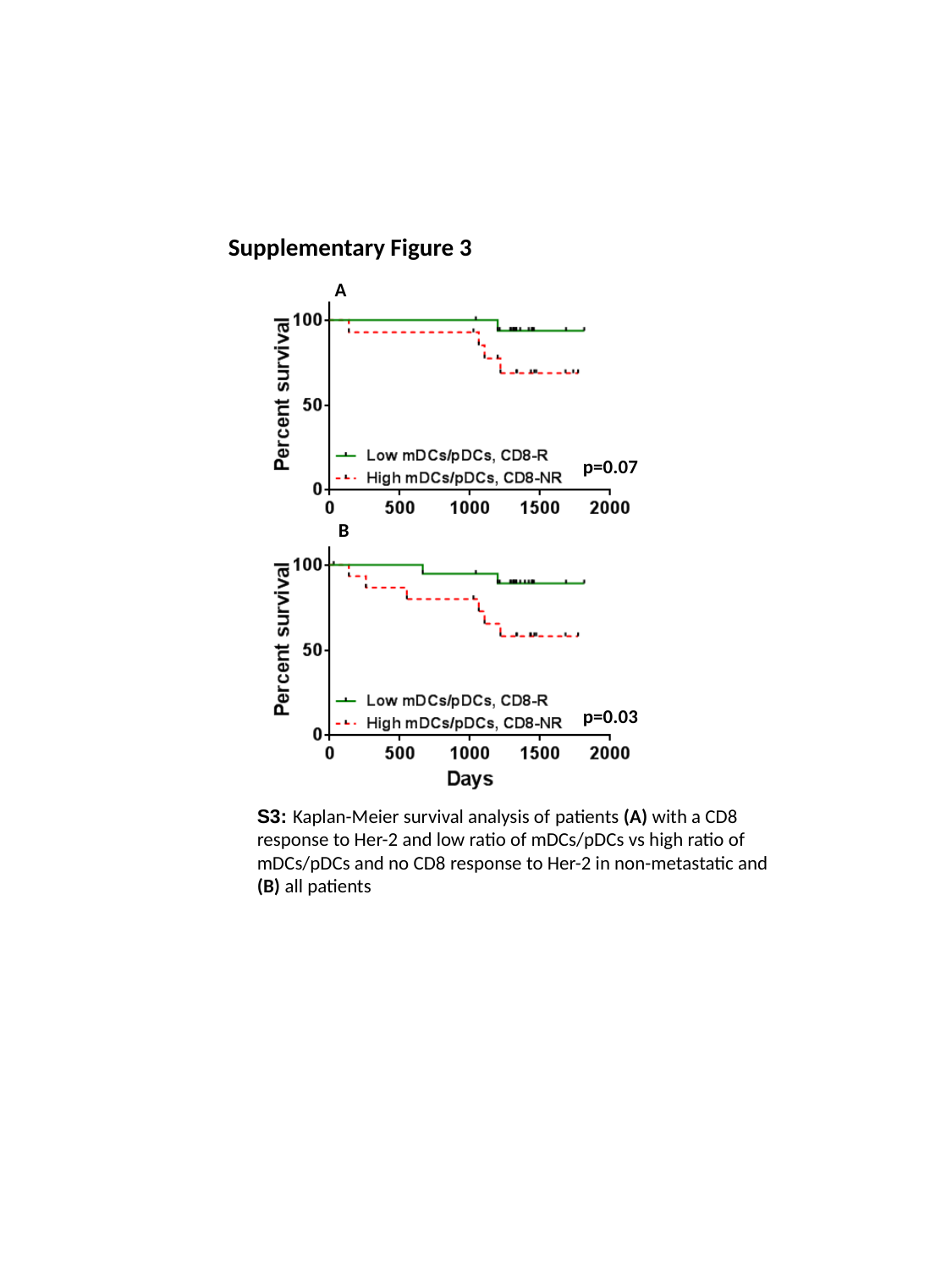

Supplementary Figure 3
A
p=0.07
B
p=0.03
S3: Kaplan-Meier survival analysis of patients (A) with a CD8 response to Her-2 and low ratio of mDCs/pDCs vs high ratio of mDCs/pDCs and no CD8 response to Her-2 in non-metastatic and (B) all patients

## Slide 4
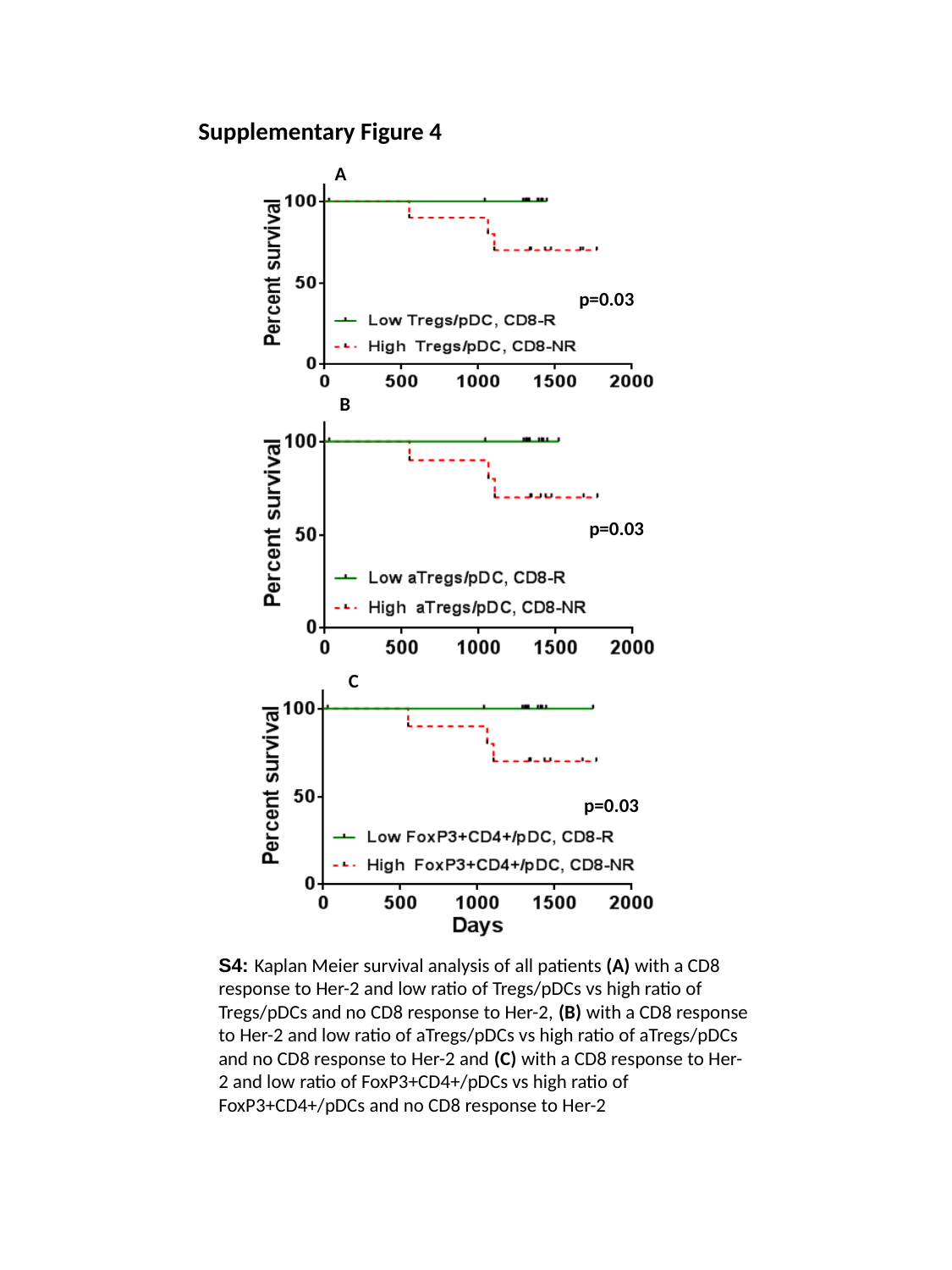

Supplementary Figure 4
A
p=0.03
B
B
p=0.03
p=0.03
C
C
p=0.03
p=0.03
S4: Kaplan Meier survival analysis of all patients (A) with a CD8 response to Her-2 and low ratio of Tregs/pDCs vs high ratio of Tregs/pDCs and no CD8 response to Her-2, (B) with a CD8 response to Her-2 and low ratio of aTregs/pDCs vs high ratio of aTregs/pDCs and no CD8 response to Her-2 and (C) with a CD8 response to Her-2 and low ratio of FoxP3+CD4+/pDCs vs high ratio of FoxP3+CD4+/pDCs and no CD8 response to Her-2
