# Supplementary material for: Prognostic impact of high levels of circulating plasmacytoid dendritic cells in breast cancer
Source: J Transl Med. 2016 May 28;14:151. doi: 10.1186/s12967-016-0905-x (PMC4884426; doi:10.1186/s12967-016-0905-x)
Supplement: Supplementary file 2 — 10.1186/s12967-016-0905-x Patients clinico-pathological parameters. [file 12967_2016_905_MOESM2_ESM.docx]

**Supplementary table 1: Patients clinico-pathological parameters**

| Clinico pathological parameters | (n=75) |
| --- | --- |
| Age range (Years) | 28-87 |
| Median age (Years) | 69 |
| **Tumor stage** | No. of patients |
| 0 | 4 |
| 1 | 30 |
| 2 | 19 |
| 3 | 12 |
| 4 | 7 |
| Unknown | 3 |
| **Tumor size** |  |
| Tis | 4 |
| T0 | 1 |
| T1 | 39 |
| T2 | 21 |
| T3 | 4 |
| T4 | 5 |
| Unknown | 1 |
| **Nodal status** |  |
| No | 41 |
| N1 | 16 |
| N2 | 7 |
| N3 | 4 |
| Unknown | 7 |
| **Metastasis** |  |
| M0 | 65 |
| M1 | 7 |
| Unknown | 3 |
| **Receptor status** |  |
| Triple Negative | 13 |
| ER+ | 58 |
| ER status unknown | 2 |
| PR+ | 53 |
| PR status unknown | 2 |
| Her-2: 2+ | 9 |
| Her-2 status unknown | 4 |
| **Treatments** |  |
| Adj.chemotherapy | 27 |
| Adj.radiotherapy | 57 |
| Adj endocrine therapy | 54 |
